# Supplementary material for: High-Throughput Screening Method Using Escherichia coli Keio Mutants for Assessing Primary Damage Mechanism of Antimicrobials
Source: Microorganisms. 2024 Apr 14;12(4):793. doi: 10.3390/microorganisms12040793 (PMC11051750; doi:10.3390/microorganisms12040793)
Supplement: Supplementary file 1 [file microorganisms-12-00793-s001.zip › microorganisms-2953931-supplementary.pdf]

Supplementary Figure S1. Characterization of AgNPs suspension: a) the impact of the increase of the NPs size distribution at different NPs concentrations by DLS analysis, b) photographs of the AgNPs suspensions using different dispersant agents (800 µg/mL) after 1 hour of ultrasound sonication (2 min, 60% amplitude); c) UV-VIS spectra of AgNPs suspension after 30, 60, 90, and 120 min of ultrasound sonication; d) TEM micrograph of Gram-negative bacteria in contact with 400 µg/mL of AgNPs.

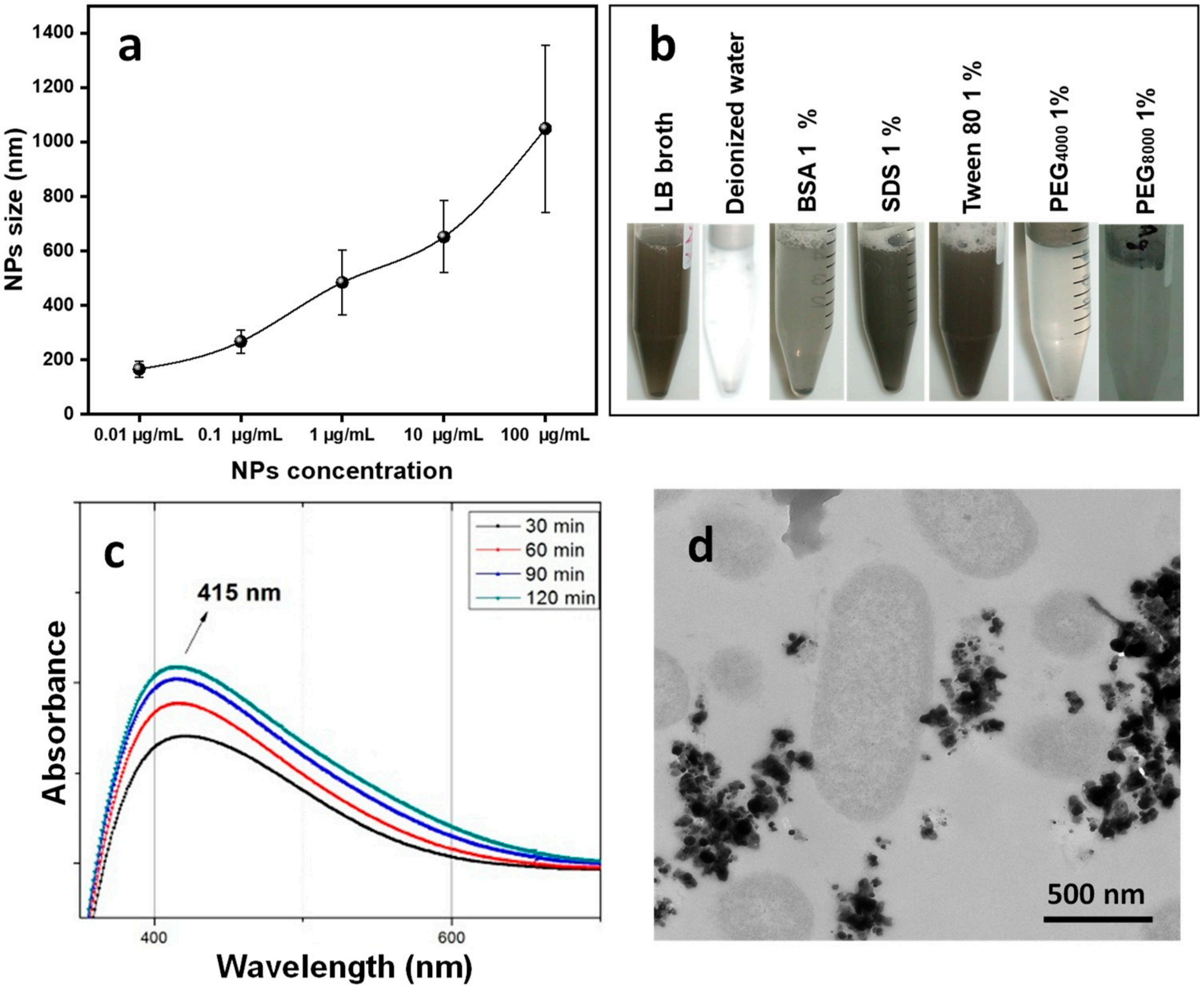
a
NPs size (nm)
NPs concentration
0.01 μg/mL
0.1 μg/mL
1 μg/mL
10 μg/mL
100 μg/mL
b
LB broth
Deionized water
BSA 1 %
SDS 1 %
Tween 80 1 %
PEG4000 1%
PEG8000 1%
c
415 nm
30 min
60 min
90 min
120 min
Absorbance
Wavelength (nm)
d
500 nm

Supplementary Figure S2. Complementary to the results of the Δ*recA* mutant strain, DH5α cells were challenged with silver NPs since this strain carries a RecA1 mutant protein. The conditions were the same as for the BW25113 parental strain. The experiment was conducted in independent triplicate assays, including the BW25113 parental strain.

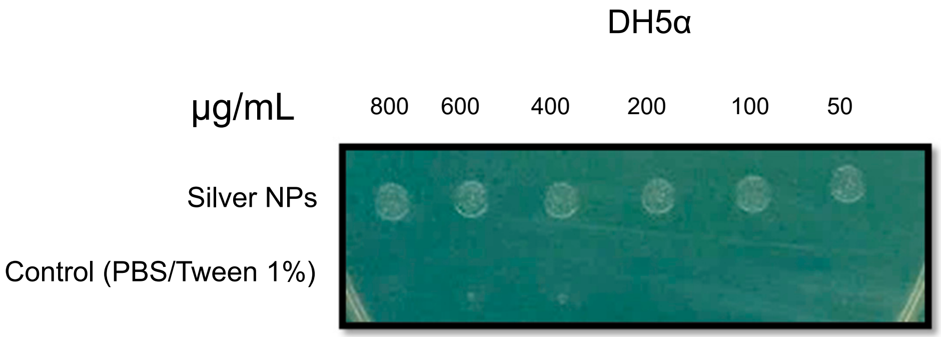
DH5α
μg/mL
800
600
400
200
100
50
Silver NPs
Control (PBS/Tween 1%)
